# Application of the Protein Maker as a platform purification system for therapeutic antibody research and development

**DOI:** 10.1016/j.csbj.2016.06.001

**Published:** 2016-06-16

**Authors:** Geneviève Hélie, Marie Parat, Frédéric Massé, Cory J. Gerdts, Thomas P. Loisel, Allan Matte

**Affiliations:** a Protein Purification, Human Health Therapeutics, National Research Council Canada, 6100 Royalmount Ave. Montreal, QC H4P 2R2, Canada; b Primary Assays, Human Health Therapeutics, National Research Council Canada, 6100 Royalmount Ave. Montreal, QC H4P 2R2, Canada; c Protein BioSolutions Inc., Suite 280, 401 Professional Drive, Gaithersburg, MD, 20879, USA

**Keywords:** CHO, Chinese Hamster Ovary, DPBS, Dulbecco's phosphate buffered saline, HC, IgG heavy chain, HCP, host cell protein, IMAC, immobilized metal ion affinity chromatography, LC, IgG light chain, mAb, monoclonal antibody, OAA, one-armed antibody, Parallelized protein purification, Antibody, Protein A, Protein G, Hybridoma, Process development

## Abstract

Within the research and development environment, higher throughput, parallelized protein purification is required for numerous activities, from small scale purification of monoclonal antibodies (mAbs) and antibody fragments for *in vitro* and *in vivo* assays to process development and optimization for manufacturing. Here, we describe specific applications and associated workflows of the Protein Maker liquid handling system utilized in both of these contexts. To meet the requirements for various *in vitro* assays, for the identification and validation of new therapeutic targets, small quantities of large numbers of purified antibodies or antibody fragments are often required. Reducing host cell proteins (HCP) levels following capture with Protein A by evaluating various wash buffers is an example of how parallelized protein purification can be leveraged to improve a process development outcome. Stability testing under various conditions of in-process intermediates, as an example, the mAb product from a clarified harvest, requires parallelized protein purification to generate concurrent samples for downstream assays. We have found that the Protein Maker can be successfully utilized for small-to-mid scale platform purification or for process development applications to generate the necessary purified protein samples. The ability to purify and buffer exchange up to 24 samples in parallel offers a significant reduction in time and cost per sample compared to serial purification using a traditional FPLC system. By combining the Protein Maker purification system with a TECAN Freedom EVO liquid handler for automated buffer exchange we have created a new, integrated platform for a variety of protein purification and process development applications.

## 1 Introduction

Purification of antibodies and antibody fragments are key activities in the generation of critical reagents for various *in vivo* and *in vitro* assays as part of biotherapeutic lead identification and process development. Often, it is necessary to purify large numbers of antibodies with milligram yield, relatively quickly and at minimal cost. Various strategies are available to achieve such purification outcomes, and can involve to various extents both automated and manual methods [1], [2]. While parallelized purification methods yielding sub-milligram quantities of pure proteins based on packed columns, 96-well plates containing small quantities of chromatographic resins or ligands immobilized to the surfaces of membranes have been developed, there are relatively fewer options available for generating purified quantitates of protein in the intermediate (5–100) milligram scale. A few examples of customized solutions to this problem exist, involving integration of existing purification platforms such as the ÄKTA Purifier with a CETAC autosampler [3], ÄKTA Pure [4] or liquid handling robotics [5] have been reported. Other solutions include the design and fabrication of customize robotics platform, including the Protein Expression and Purification Platform [6]. While some commercial instruments for purification of small quantities of protein have been developed, such as the QIAcube for purification of His-tagged proteins [7], there are few examples of commercial instruments that can be utilized for platform purification at milligram scale.

In the context of process development applications, various commercially available scale-down protein purification products have been developed, including Predictor plates (GE) and Robo-columns (GE and Atoll Bio). While very useful for early-stage screening of various chromatographic conditions, the maximum size of the columns possible in these platforms (600 μL bed volume) results in a considerable gap in the scale between screening and further optimization of process conditions. Some examples of higher throughput, automated solutions to purification process development have been reported [8], [9]. While automated, sequential purification of samples is possible using a chromatography system connected to an auto-sampler, this cannot be parallelized using a single instrument, thereby reducing the possible number of samples processed.

A specific instrument which has been designed around accomplishing the task of parallelized, medium scale purification is the Protein Maker system, originated by Emerald BioStructures [10] and subsequently developed and marketed by Protein BioSolutions. The Protein Maker is an automated protein purification platform designed for purification of feed volumes of various sizes, from ~ 10 mL to 1 L (~ 1 mg to 100 mg) or more utilizing up to 24 chromatography columns, each with an independent flow path. The main components of the system are (i) the syringe pumps with the associated 9-port valve, mixing syringe and sample lines, which together form the initial portion of the flow path, (ii) the column gantry, columns and associated tubing from the syringe pumps, which form the subsequent portion of the flow path and (iii) the deck, which contains up to 19 positions for SBS format plates and a dedicated waste position.

While purification of a variety of proteins from any number of sources is in principle possible with the instrument, the focus herein are examples of purification of antibodies and their fragments generated from mammalian expression systems. We have utilized the Protein Maker as a key component of a platform purification system that integrates automated buffer exchange implemented on a TECAN Freedom EVO liquid handler. This protein purification platform can be used for both parallelized, small-medium scale purification of antibodies and their fragments, as well in various process development applications.

## 2 Materials and methods

### 2.1 Antibody production

Murine IgG samples were produced in hybridoma culture in IMDM supplemented with 10% heat-inactivated FBS and mouse IL-6 by a procedure previously described [11]. For some antibodies, cultures were performed transiently in Chinese Hamster Ovary (CHO) cells as previously described [12]. Productions were harvested by centrifugation or filtration (0.22 μm or 0.45 μm) and IgG containing supernatants stored at 4 °C until purified.

### 2.2 Purification of mAbs and Fabs

For development of Protein Maker purification methods, protein samples were purified using 1 mL HiTrap columns (GE Life Sciences), including Protein G HP, MabSelect SuRe™ (Protein A) and Ni Sepharose Excel™ mounted on an ÄKTA Purifier 10/100 system. Chromatographic profiles were monitored at 280 nm. Columns were equilibrated in Dulbecco's Phosphate Buffered Saline Solution (DPBS, HyClone Laboratories), the sample applied at the appropriate residence time (1 min for Ni Sepharose Excel or 3 min for MabSelect SuRe or Protein G HP) and a portion of the flow-through fraction collected for subsequent non-reducing SDS-PAGE analysis. Columns were washed with DPBS and bound proteins eluted with two column volumes (CV) of sodium-citrate buffer pH 3.6 (MabSelect SuRe), two CV of 100 mM glycine-HCl pH 2.6 (Protein G HP) or one CV of DPBS with 500 mM imidazole pH 8 (IMAC purification). For proteins eluted from Protein A or Protein G columns, samples were pH adjusted using 1 M solutions of sodium HEPES or Tris–HCl buffer to a final pH of 6–7.

Platform, parallelized purification experiments were performed using the Protein Maker running the Protein Maker v2.0 software (Protein BioSolutions). Purification runs were performed using the 1 mL HiTrap columns and the chromatography conditions (residence time, column washing and sample elution) established using the ÄKTA purification system. Sample and buffer lines were cleaned in place with 0.5 M NaOH and equilibrated in DPBS or appropriate buffer solutions. During purification, a portion of the flow-through fraction was collected for subsequent non-reducing SDS-PAGE analysis. Protein samples were eluted in three steps, consisting of a pre-elution volume, elution volume and post-elution volume. Protein concentration measurements (A_280_ nm) on these fractions were used to establish the final pooled sample.

### 2.3 Process development for mouse IgG2a purification

The Protein Maker system was used to purify in parallel five murine IgG2a samples from mouse Hybridomas using MabSelect SuRe and Protein G HP 1 mL HiTrap columns. For each mouse IgG2a, 20–22 mL of supernatant (~ 0.5 to 2 mg of mouse IgG2a, depending on titer) was purified on either column using the purification method described above. For protein G purifications, elution was performed in two steps, first with 100 mM citrate buffer pH 3.6 and then with 100 mM glycine-HCl buffer pH 2.6. Elution fractions were neutralized using 1 M Tris. The quantity of IgG2a contained in elution fractions were determined based on A_280_ nm. For protein G purification, the quantity of IgG2a obtained from the two elution steps was summed for calculating the yield.

### 2.4 Development of a post-load wash step to improve HCP removal during protein A purification

Data were obtained with three different antibodies expressed in CHO cells. The Protein Maker system was used to perform parallel purifications using MabSelect SuRe 1 mL HiTrap columns. For each antibody, eight wash conditions were tested. For each tested condition, 10 mL of supernatant (16.3 to 17.5 mg of antibodies) were loaded at a residence time of 3 min. HiTrap columns were then washed and antibodies were eluted using 2.5 CV of 100 mM citrate buffer pH 3.0. Elution fractions were neutralized using 1 M HEPES. The quantity of antibody in elution fractions was determined by A_280_ nm. The quantity of HCP in elution fractions was measured using a CHO HCP ELISA kit (Cygnus Technologies).

### 2.5 Buffer exchange and aseptic filtration

Buffer exchange into DPBS following affinity purification was performed manually either using Zeba-spin columns (Thermo-Fisher Scientific) by centrifugation or using PD-10 desalting columns (GE Healthcare) by gravity according to the manufacturer's instructions. Alternatively, sample buffer exchange using PD Miditrap G-25 columns was automated on a TECAN Freedom EVO150® liquid handler according to gravity protocols from GE Healthcare. The Freedom EVO150® was equipped with a liquid displacement Liquid Handler (LiHa) configured with 8 channels (4 disposable tips and 4 washable tips), a Robotic Manipulator (RoMa), a Tecan Vacuum (TeVac), carriers for 11 microplates, shelf for 4 microplates, one reservoir position for elution buffer, and tip carriers for hanging tips. All tubing and components of the liquid displacement system were cleaned in-place with 0.5 M NaOH for at least 15 min and rinsed with sterile water prior to operations. A script was developed with the flexibility to process from 24 to 96 samples at once. Before starting, the storage solution from PD MidiTrap G25 columns was removed manually, the columns placed in a 24 position custom holder and the rack positioned on the Freedom EVO150® worktable. Purified protein samples from the Protein Maker were stored in 24 deep well microplate (Seahorse Bioscience). The system liquid was replaced with DPBS (HyClone Laboratories) and the script started. First, the RoMa arm brought the column racks onto the TeVac and columns were equilibrated with three bed volumes using the Freedom EVO150® system liquid (DPBS). The equilibration buffer was allowed to enter the packed bed completely and the flow-through discarded in the TeVac waste. Samples were pipetted onto the columns using disposable filter tips (Tecan) and time was allowed for them to enter the packed bed by gravity. Column racks were tapped 4 × times on the Te-Vac by the RoMa arm to remove any droplets and transported on top of a 24 deep well block for elution. The LiHa pipetted 1.5 mL of elution buffer (DPBS) to each MidiTrap, respectively, using disposable tips and the eluate containing the protein of interest was collected by gravity. Subsequent aseptic filtration was performed by centrifugation using either sterile Multiscreen 0.22 μm 96-well plates (Millipore) or deep well 0.22 μm 96-well plates (Corning) stacked with a sterile receiver plate.

### 2.6 Analytical methods

Proteins were quantitated based on A_280_ nm values obtained using a NanoDrop 2000 spectrophotometer (Thermo Scientific) and concentration values were corrected based on calculated extinction coefficients derived from the protein sequence. Non-reducing SDS-PAGE analysis was performed using 4–12% Bis-Tris NuPage gels (Novex, Thermo-Fisher scientific) and stained with Sypro Ruby protein gel stain (Thermo-Fisher Scientific) as recommended by the manufacturer. SDS-PAGE gels were imaged using a ChemiDoc MP imaging system (Bio-Rad Laboratories).

## 3 Results

### 3.1 Development and implementation of protein maker platform purification methods

**Fig. 1:**
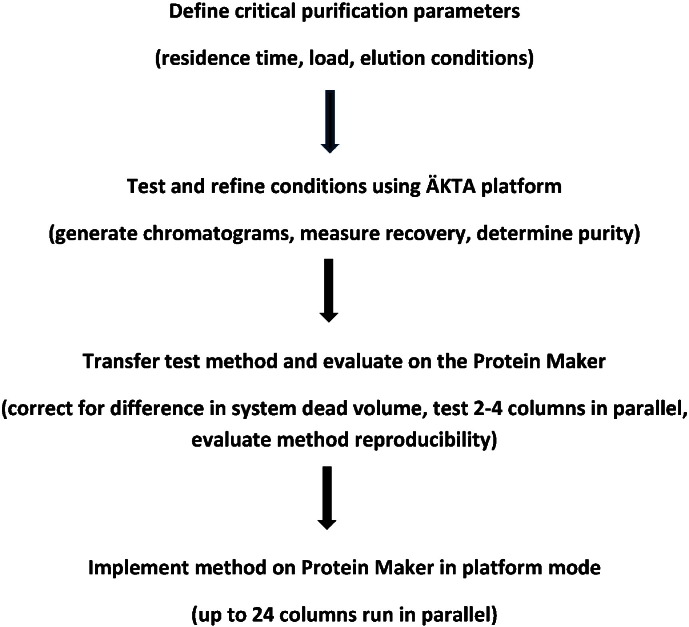
Protein Maker purification development strategy.

Our strategy for the development and implementation of affinity purification methods is summarized in Fig. 1. Key factors that influence the purification process, including residence time for product capture and elution volume are determined using the ÄKTA purification runs. The essential value of these runs is to provide the absorbance trace during the purification in order to quickly converge to appropriate starting conditions for purification. The essential features of the different affinity purification methods are similar for Protein G, Protein A and IMAC based purification method development. Overall, we observed comparable results in terms of product yield and sample purity when using the same purification method on an ÄKTA purification platform and the Protein Maker (results not shown).

A key factor in establishing the capture step is to determine the residence time to achieve optimal capture of the product on the column of interest. In the case of Protein G affinity chromatography, using a residence time of 3 min, no IgG was found in the flow-through of hybridoma-generated mAb samples as determined by non-reducing SDS-PAGE with Sypro Ruby staining. For larger volumes of feeds containing small quantities of product, it is necessary to split samples over two or more columns in order to reduce the total time required for the binding step. Using such an approach, ~ 200–250 mL of product (~ 30 mg of protein in the case of Protein A) can be passed over 1 mL columns in an overnight run.

**Fig. 2:**
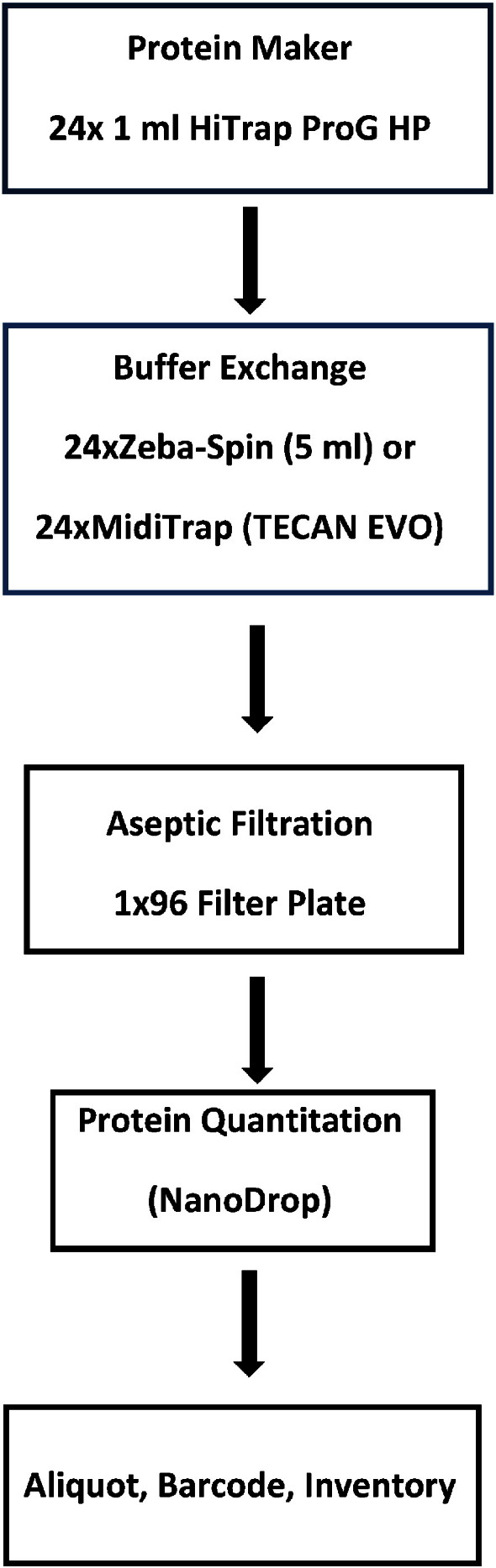
Protein G purification workflow for purification of mAbs from hybridoma supernatants.

We have found that using the approach of collecting elution fractions in three steps (pre-elution (0.5 CV), main elution (0.5–1.5 CV) and post-elution (1.5 + CV) volumes) allows for optimization of the quantity of purified product in a minimum volume, allowing for maximum product concentration. At the elution step, the practical outcome is to obtain ≥ 80% of the purified protein in a minimum volume suitable for manual or automated buffer exchange as the second step in the workflow shown in Fig. 2.

### 3.2 Platform purification applications

#### 3.2.1 Small scale purification of mAbs from hybridoma supernatants

In order to generate purified mAbs for cell-based assays, we have developed a Protein G based purification workflow using Protein G HP as the capture resin (Fig. 2). Each purification cycle permits 23 mAb samples along with one murine IgG control sample, to be purified using Protein G, buffer exchanged into DPBS and aseptically filtered prior to aliquoting in bar coded tubes for storage. The purpose of the control sample is to act as a sentinel for the purification run *via* determination of the recovery of purified protein. In principle, this sample will help to troubleshoot problems in executing the purification method, as for example, a change in column binding capacity resulting from excessive numbers of clean in place (CIP) cycles.

**Fig. 3:**
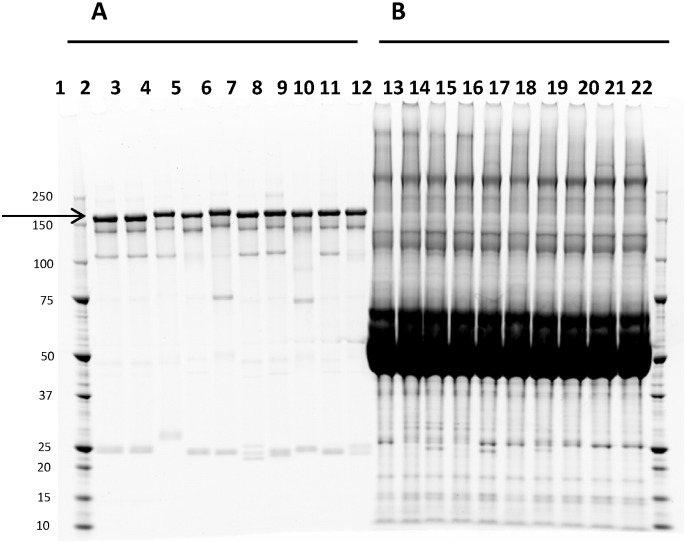
Non-reducing SDS-PAGE and Sypro Ruby staining of ten different hybridoma-produced antibodies purified using Protein G HP with the Protein Maker (A) purified mAbs, (lanes 2–11)(B) flow-through fractions (lanes 12–21), revealing no unbound mAb (arrow). The mAb species (2HC + 2LC) is indicated with an arrow, and minor species include 2HC (~ 100 kDa), half-antibody (~ 75 kDa) and free LC (~ 25 kDa) are visible for some samples. Molecular weight markers (lanes 1, 22) are shown in kDa.

In most cases, approximately 0.1–1 mg of purified mAb were obtained per hybridoma supernatant, mainly with a concentration range of approximately 0.75 to 0.95 mg/mL. Non-reducing SDS-PAGE followed by staining with Sypro Ruby, a high-sensitivity protein stain, revealed no IgG in the flow-through fraction (Fig. 3B). The purity of the resulting purified mAbs was also verified by non-reducing SDS-PAGE (Fig. 3A).

In early experiments, samples were buffer exchanged manually using Zeba-spin centrifugal columns. While this approach minimizes sample dilution, it is manually intensive, and sub-optimal within a high throughput protein purification workflow (Fig. 2). Using PD MidiTrap G-25 columns in gravity mode, we implemented an automated approach to buffer exchange samples following affinity purification, reducing the manual labor, time and cost associated with this step. A consequence of this change was increased dilution of the purified sample by ~ 1.5-fold compared to the Zeba-spin buffer exchange.

#### 3.2.2 Antibody fragments — purification of Fabs

**Fig. 4:**
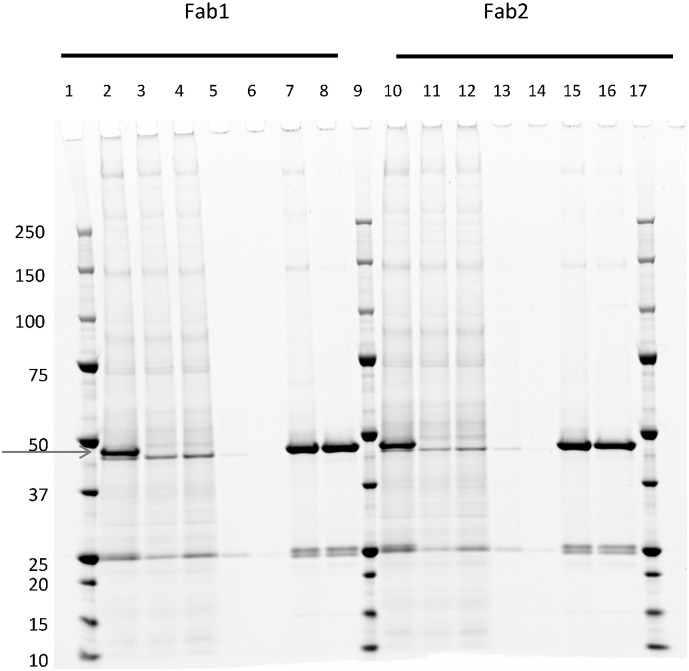
Non-reducing SDS-PAGE and Sypro Ruby staining of CHO-produced and IMAC purified Fab samples using the Protein Maker. Lanes 1, 9, 17, molecular weight markers, in kDa; lanes 2, 10, CHO supernatants of expressed Fabs; Lanes 3,4,11,12, flow-through fractions; lanes 5,6,13,14, wash fractions; lanes 7,8,15,16, elution fractions. The ~ 50 kDa species in the F/T fraction represents light chain (LC) dimers.

We applied the Protein Maker platform to the purification of a series of his-tagged Fab constructs generated for *in vitro* studies. Other than the buffer composition for column washing and sample elution, the overall strategy is similar to that applied to Protein G or Protein A purification methods. Using this approach, up to 20 mg of purified Fab from ~ 200 mL CHO culture has been obtained. Recoveries post-buffer exchange are similar to that obtained for mAbs purified using Protein G or Protein A. The purity of representative Fab samples as determined by non-reducing SDS-PAGE is shown in Fig. 4.

### 3.3 Process development applications

#### 3.3.1 Evaluation of protein A *vs* protein G resin for purification of murine IgG2a

**Fig. 5:**
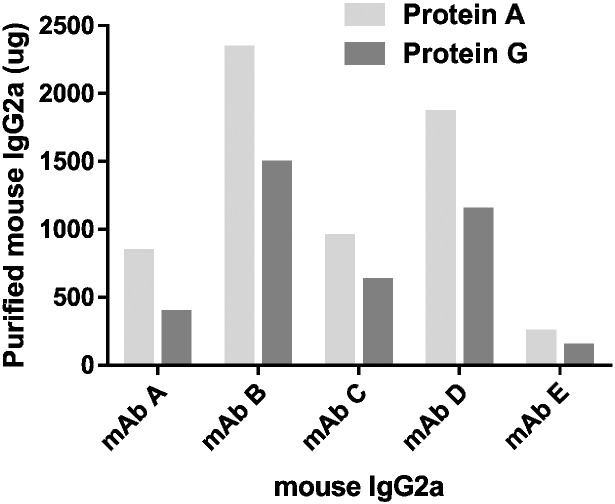
Quantities of mouse IgG2a (μg) purified from mouse hybridoma supernatants using either Protein A or Protein G.

Protein G resin is typically considered as the first choice to purify antibodies produced from mouse Hybridomas, especially when the IgG subclass is unknown. Indeed, Protein G has strong affinity for all mouse IgG subclasses, whereas Protein A has strong affinity for only certain subclasses, specifically IgG2a and IgG2b. For purification of mouse IgG2a, the choice of one resin *vs* the other is not straightforward. According to some manufacturers, mouse IgG2a has strong binding affinity for both Protein A and Protein G, however, protein A columns have a higher binding capacity and could be a better choice especially for larger scale purification. To determine which of Protein A or Protein G is the better choice to purify mouse IgG2a, we used the Protein Maker to purify five mouse IgG2a samples in parallel on these columns. The total quantity of mouse IgG2a obtained with either resin following purification is summarized in Fig. 5. For all tested mouse IgG2a, the quantity of purified protein obtained from Protein A was at least 50% higher than those obtained from Protein G purification.

#### 3.3.2 Development of a post-load wash step to improve HCP removal during protein A purification

**Fig. 6:**
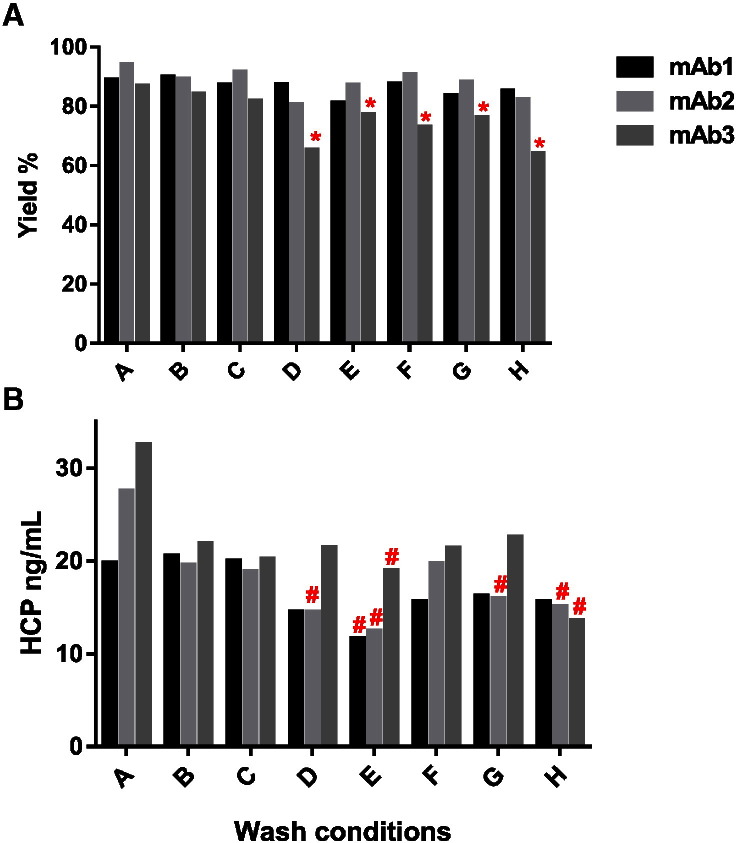
Effect of wash buffers on (A) purification yields and (B) HCP levels. Wash conditions — A: PBS; B: 100 mM Citrate, 50 mM NaCl pH 5.0; **C**: 50 mM Tris, 100 mM Arginine, 50 mM NaCl pH 7.0; D: 50 mM Tris, 100 mM Arginine, 50 mM NaCl pH 8.0; E: 50 mM Tris, 100 mM Arginine, 50 mM NaCl pH 9.0; F: 10 mM Phosphate, 100 mM Arginine, 50 mM NaCl pH 7.0; G: 10 mM Phosphate, 100 mM Arginine, 50 mM NaCl pH 8.0; H: 10 mM Phosphate, 100 mM Arginine, 50 mM NaCl pH 9.0. *: Yields below 80% — #: HCP reduction *vs* PBS greater than 1.7-fold.

Removing impurities post-Protein A chromatography still represents a significant challenge to purification process development in order to achieve the required drug substance specifications suitable for patient administration (HCP < 100 ppm). Using a post-load wash step is a key means to achieve HCP clearance, since it has been demonstrated that HCP associates with antibodies and co-elute during the elution step. Basic pH and wash additives such as arginine seem to improve HCP removal during the Protein A chromatography wash step by disrupting interactions between the antibody and the HCPs [13]. Here we evaluated both Tris and phosphate-based arginine wash buffers at different pH values (7 to 9) in comparison to conventional wash buffer (DPBS or citrate pH 5.0) using 1 mL HiTrap MabSelect SuRe columns. These various wash conditions (Table 1) were tested in parallel with 3 different antibodies (CHO supernatants) using the Protein Maker. Yield and HCP levels obtained for each wash condition are presented in Fig. 6. The use of basic wash buffers containing arginine showed improved HCP removal (1.7 to 2.4-fold) compared to the conventional DPBS wash for the three antibodies tested, with detrimental effects on purification yields only for one antibody (mAb3).

#### 3.3.3 Stability hold of clarified harvest for a mAb

In a series of experiments, a clarified mAb harvest was held at either 2–8 °C or 19–23 °C and sampled at various time points corresponding to t = 0, 1, 2, 3, 5 and 7 days before purification using MabSelect SuRe followed by buffer exchange to DPBS in order to evaluate changes in the product using an analytical assay panel. The purification performance at time points, t = 0 and t = 7 days, is shown in Table 2. Purification recoveries based on the measured product titer were at least 85%. Analysis of charge variants and glycosylation profiles at each time point at either temperature revealed a significant decrease in acidic charge variants upon storage at 19–23 °C, corresponding to a drop in the sialic acid content of the mAb (results not shown). This assessment made it possible to define the manufacturing hold time duration necessary for process control during GMP manufacturing.

## 4 Discussion

During early-stage therapeutic antibody R&D projects, it is often necessary to purify large numbers of samples that will be used as reagents for *in vivo* or *in vitro* screening assays. The nature of the purification strategy employed is determined by the quantity and final concentration of purified protein required; the number of samples that need to be purified per unit time (throughput), the supernatant volume and initial product titer, as well as the availability, if any, of automated liquid handling instrumentation. While clearly there is more than one possible solution to achieve the purification objectives, we selected the Protein Maker as a platform using HiTrap columns, including Protein G (Protein G HP), Protein A (MabSelect SuRe) and IMAC (Ni Sepharose Excel), and have applied these to feeds from either hybridoma or CHO supernatants ranging from 10 mL to 200 mL volumes. A key outcome has been for purification of small quantities (< 1 mg) of purified mAbs using the Protein G method described here, with greater than 1000 mAbs purified and utilized as reagents for *in vitro* screening assays.

For the development of platform-based purification methods on the Protein Maker, several parameters that influence the overall purification outcome required evaluation. One of the most critical parameters is the residence time used in the binding step, as most of the overall time required to execute the method involves binding of the product to the packed bed. Through several purification campaigns utilizing Protein G HP with murine IgG's, we have established that a three-minute residence time ensures capture of the product, as revealed by non-reducing SDS-PAGE analysis. Another critical parameter is to establish the elution volume range for the product. The elution volume will depend on the column volume and the quantity of product bound to the packed bed, with larger elution volumes being required as one approaches the dynamic binding capacity limit of the column. The optimal elution volume is a balance between the desired product concentration, yield and recovery of the purified product. One rule of thumb would be to establish the elution volume such that ≥ 80% of the purified protein is captured in one fraction.

In many antibody affinity purification work-flows, purification is followed by buffer exchange into a formulation buffer, including PBS, in order to minimize protein aggregation as a result of unfavorable conditions of pH or ionic strength. While it is possible to perform the buffer exchange step in high-throughput mode using 96-well plates [14], this only applies to samples having a small volume, typically less than 130 μL. Initially, we performed this step in a more manually intensive manner using Zeba-spin buffer exchange columns. In order to increase the throughput and operational efficiency of the buffer exchange step, a more automated approach was developed using PD MidiTrap G-25 columns on a TECAN Freedom EVO 150 liquid handling system. The integration of an automated buffer exchange step in a 24-sample format, the same format as the Protein Maker, increases the overall sample throughput, decreases manual manipulations and enhances the consistency of results.

In addition to platform-based purification of full-size IgG's, there is often a requirement to purify IgG fragments, including Fabs and scFv's, as well as to evaluate the effectiveness of different affinity purification resins for purification of the same product. For purification of murine IgG2a, the choice of Protein A *vs* Protein G purification requires experimental verification prior to establishing the final process to be used. By performing all of the purification experiments in parallel using the Protein Maker, a few hours were sufficient to determine that protein A is the better choice for purification of murine IgG2a samples. Indeed, the quantity of purified protein obtained from Protein A was at least 50% higher than those obtained from Protein G purification, although in the absence of additional data we cannot offer a definitive explanation for this result. Executing the same experiment using one ÄKTA purification system would require approximately five-fold more time than performing the purification experiments in parallel with the Protein Maker. It is noteworthy that these 5 antibodies were subsequently purified at larger (2 L) scale using protein A resin with greater than 80% recovery for four out of the five antibodies processed. In the purification scheme presented here, Fabs are purified *via* his-tags using IMAC, although Fab purification can be achieved in some instances using protein A and more commonly using the CH1 domain of the Heavy Chain (HC) or the conserved domain of the Light Chain (LC) as capture modes. Other therapeutic antibody formats, including one-armed antibodies, hybrids, bi-specifics and various Fc-fusion molecules can also be purified in platform mode with the appropriate affinity resin. Examples of readily available affinity chromatography resins and how they can be applied to various antibody purification requirements is summarized in Table 3.

Both the post-load wash step for optimization of HCP removal from Protein A as well as the clarified harvest hold stability study offer examples of how the Protein Maker can be effectively utilized in the context of protein purification process development. Minimizing host cell protein levels at the Protein A capture step through modification of wash buffer composition can improve the purification process [13], [15], [16]. One cycle of parallelized purification using the Protein Maker, requiring approximately 3 h, was sufficient to purify 24 samples (eight different wash conditions for three different antibodies). Executing the same experiment using one ÄKTA purification system (*i.e.* 24 sequential purifications on an ÄKTA Purifier) would have required approximately 37.5 h. In this experiment, the Protein Maker not only increased purification throughput by 12.5-fold but also permitted purification of samples in an unbiased manner by having the capacity to purify all of the samples at the same time. Indeed, under conditions where a mAb is unstable in the clarified harvest, the ability to purify multiple samples in parallel offers a distinct advantage over serial purification using conventional purification equipment, minimizing misinterpretation of data due to sample degradation. As the effectiveness of optimized wash buffers and their effect on purification yields seem to vary depending on the antibody, the same wash conditions cannot be used for all samples. Rather, post-load wash conditions should be optimized for each antibody as part of purification process development. Due to its ability to process multiple samples in parallel, the Protein Maker represents a preferred instrument for performing wash buffer screening. Moreover, as multiple wash conditions can be evaluated in parallel, it is possible to minimize variability in sample handling that could bias data interpretation.

Therapeutic antibody products often pose various challenges during the development of upstream and downstream processes, including degradation, modification of Fc or Fd glycans, reduction of intramolecular disulfides, deamidation of Asn and Gln residues, as well as aggregation [17]. In order to de-risk the overall process, an important component of the transition between upstream and downstream processing is to understand the stability of the product post-clarification but prior to the initial capture purification step. The removal of sialic acid from mAbs by the action of extracellular CHO sialidase is an example of the kinds of post-production, pre-purification modifications that are possible [18]. The utility of the Protein Maker in this application is its ability to purify several samples in parallel, for example, if the clarified harvest is to be held at various temperatures or at different pH values, or if multiple harvests are to be tested in parallel and purified under the same controlled conditions.

## 5 Conclusions

We have found the Protein Maker to be a versatile tool for a number of purification problems, ranging from small scale mAb and Fab purification to various applications in process development. We have developed platform purifications methods using Protein G, Protein A and IMAC and have applied these to several projects. Use of the Protein Maker and TECAN Freedom EVO150 together has resulted in reducing both the required personnel time and creation of an integrated workflow that includes both protein purification and automated buffer exchange in a 24-sample format. Future implementation of the UV monitoring capability will add an additional dimension to the capabilities of this instrument, permitting 24-channel monitoring of the protein absorbance signal, thereby expediting utilization of the instrument for various process development applications.

## Figures and Tables

**Table 1:** Wash conditions tested for Protein A post-load HCP removal**.**

	Wash 1 (5 CV)	Wash 2 (5 CV)	Wash 3 (5 CV)
1	PBS	PBS	PBS
2	PBS	100 mM Citrate, 50 mM NaCl pH 5.0	PBS
3	PBS	50 mM Tris, 100 mM Arginine, 50 mM NaCl pH 7.0	PBS
4	PBS	50 mM Tris, 100 mM Arginine, 50 mM NaCl pH 8.0	PBS
5	PBS	50 mM Tris, 100 mM Arginine, 50 mM NaCl pH 9.0	PBS
6	PBS	10 mM Phosphate, 100 mM Arginine, 50 mM NaCl pH 7.0	PBS
7	PBS	10 mM Phosphate, 100 mM Arginine, 50 mM NaCl pH 8.0	PBS
8	PBS	10 mM Phosphate, 100 mM Arginine, 50 mM NaCl pH 9.0	PBS

**Table 2:** Chromatographic Performance Table — MabSelect SuRe purification, clarified harvest stability hold study for t = 0 and t = day 7. The global process purification yield is calculated as the ratio of the final yield over the initial load on the column.

Storage temperature (°C)	Time point (Day)	HiTrap Mab Select SuRe				PD-10 buffer exchange and aeptic filtration			Global process yield (%)
		Load		Elution		Load	Final		
		Volume (mL)	Quantity (mg)	Quantity (mg)	Step yield (%)	quantity (mg)	quantity (mg)	Step yield (%)	
N/A	0	50	16.6	14.8	89	13.6	13.6	99.9	82.0
2–8 °C	7	50	16.5	14.0	85	12.8	12.6	98.7	76.6
19–23 °C	7	50	16.6	15.3	92	14.0	13.7	97.5	82.6

**Table 3:** Possible platform affinity purification modes for antibodies and antibody fragments using the Protein Maker**.**

Format	Isotype	Species	Capture mode	Resin
mAb, bispecific	IgG1, IgG2, IgG4	Human	Fc	Protein A
mAb, bispecific	IgG2	Murine	Fc	Protein A
mAb, bispecific	IgG1, IgG2, IgG3, IgG4	Human	Fc	Protein G
mAb, bispecific	IgG1, IgG2, IgG3	Murine	Fc	Protein G
OAA	N/A	Human	Fc	Protein A, G
scFv		Human	LC (V_L_)	Protein L
Hybrid		Human	Fc	Protein A, G
Hybrid		Human	HC (CH1)	CH1
Hybrid		Human	LC (κ, C_L_)	Kappa-Select
Fab		Human	HC (CH1)	CH1
Fab		Human	LC (κ, C_L_)	Kappa-Select
Fab		Human	LC (λ, C_L_)	Lambda-Fab Select

## References

[1] Cummins E., Luxenberg D.P., McAleese F., Widom A., Fennell B.J., Damanin-Sheehan A. (2008). A simple high-throughput purification method for hit identification in protein screening. J Immunol Methods.

[2] Su B., Hrin R., Harvey B.R., Wang Y.J., Ernst R.E., Hampton R.A. (2007). Automated high-throughput purification of antibody fragments to facilitate evaluation in functional and kinetic based assays. J Immunol Methods.

[3] Yoo D., Provchy J., Park C., Schulz C., Walker K. (2014). Automated high-throughput protein purification using an AKTApurifier and a CETAC autosampler. J Chromatogr A.

[4] Holenstein F., Eriksson C., Erlandsson I., Norrman N., Simon J., Danielsson A. (2015). Automated harvesting and 2-step purification of unclarified mammalian cell-culture broths containing antibodies. J Chromatogr A.

[5] Alm T., Steen J., Ottosson J., Hober S. (2007). High-throughput protein purification under denaturing conditions by the use of cation exchange chromatography. Biotechnol J.

[6] Gonzalez R., Jennings L.L., Knuth M., Orth A.P., Klock H.E., Ou W. (2010). Screening the mammalian extracellular proteome for regulatorsof embryonic human stem cell pluropotency. Proc Natl Acad Sci U S A.

[7] McGraw J., Tatipelli V.K., Feyijinmi O., Traore M.C., Eangoor P., Lane S. (2014). A semi-automated method for purification of milligram quantities of proteins on the QIAcube. Protein Expr Purif.

[8] Kelley B.D., Switzer M., Bastek P., Kraimarczyk J.F., Molnar K., Yu T. (2008). High-throughput screening of chromatographic separations: IV. Ion-exchange. Biotechnol Bioeng.

[9] Teeters M., Bezila D., Alread P., Velayudhan A. (2008). Development and application of an automated, low-volume chromatography system for resin and condition screening. Biotechnol J.

[10] Smith E.R., Begley D.W., Anderson V., Raymond A.C., Haffner T.E., Robinson J.I. (2011). The Protein Maker: an automated system for high-throughput parallel purification. Acta Crystallogr F.

[11] Yokoyama W.M., Li Z. (2001). Monoclonal antibody supernatant and ascites fluid production. Curr Protoc Immunol.

[12] Raymond C., Robotham A., Spearman M., Butler M., Kelly J., Durocher Y. (2015). Production of α2,6-sialylated IgG1 in CHO cells. MAbs.

[13] Chollangi S., Parker R., Singh N., Li Y., Borys M., Li Z. (2015). Development of robust antibody purification by optimizing protein-A chromatography in combination with precipitation methodologies. Biotechnol Bioeng.

[14] Ying W., Levons J.K., Carney A., Gandhi R., Vydra V., Rubin A.E. (May 12 2015). Semiautomated sample preparation for protein stability and formulation via buffer exchange. J Lab Autom.

[15] Aboulaich N., Chung W.K., Thompson J.H., Larkin C., Robbins D., Zhu M. (2014). A novel approach to monitor clearance of host cell proteins associated with monoclonal antibodies. Biotechnol Prog.

[16] Sisodiya V., Lequieu J., Rodriguez M., McDonald P., Lazzareschi K.P. (2012). Studying host cell protein interactions with monoclonal antibodies using high throughput protein A chromatography. Biotechnol J.

[17] Vasquez-Ray M., Lang D.A. (2011). Aggregates in monoclonal antibody manufacturing processes. Biotechnol Bioeng.

[18] Gramer M.J., Goochee C.F., Chock V.Y., Brousseau D.T., Sliwkowski M.B. (1995). Removal of sialic acid from a glycoprotein in CHO cell culture supernatant by action of an extracellular CHO cell sialidase. Biotechnology (N Y).

